# Supplementary material for: Identification of photoperiod-induced specific miRNAs in the adrenal glands of Sunite sheep (Ovis aries)
Source: Front Vet Sci. 2022 Jul 22;9:888207. doi: 10.3389/fvets.2022.888207 (PMC9354845; doi:10.3389/fvets.2022.888207)
Supplement: Supplementary file 1 [file Data_Sheet_1.ZIP › Supplementary file/miRDeep2 file.zip!/miRDeep2 file/Novel_Structure/LP42A3/Novel_84.pdf]

Provisional ID	:	NC_019463.2_9181
Score total	:	48.2
Score for star read(s)	:	3.9
Score for read counts	:	40.4
Score for mfe	:	2.3
Score for randfold	:	1.6
Score for cons. seed	:	
Total read count	:	91
Mature read count	:	88
Loop read count	:	0
Star read count	:	3

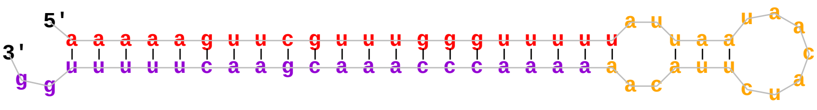

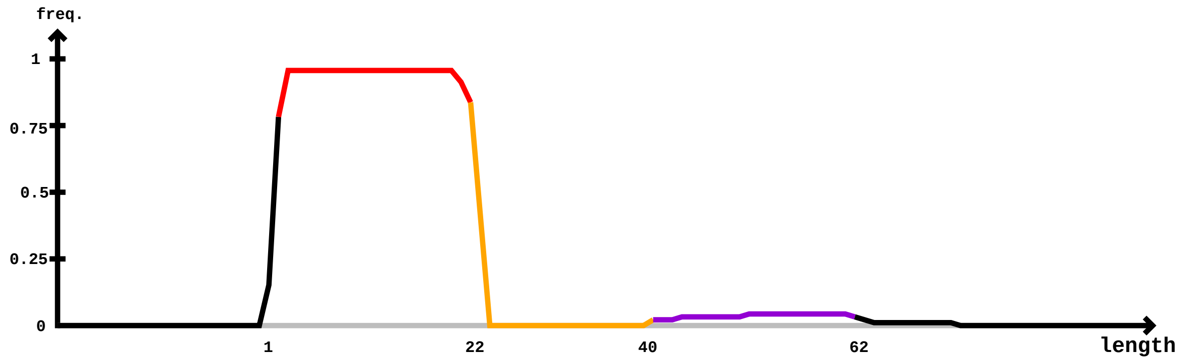

Mature   Star

```
5'- ugaaaagguauuggguuggccaaaaaguucguuuggguuuuuauuaauaacaucuuacaaaaaacccaaacgaacuuuuuggcccaacccaguauuugagaugauuacugcu   -3'   obs
    ugaaaagguauuggguuggccaaaaaguucguuuggguuuuuauuaauaacaucuuacaaaaaacccaaacgaacuuuuuggcccaacccaguauuugagaugauuacugcu         exp
    .....(((((((((((((((((((((((((((((((((((((..(((.......)))..))))))))))))))))))))))).))))))))))))))...............   reads   mm   sample
    ....................caaaaaguucguuuggguuu........................................................................   1       0    seq
    ....................caaaaaguucguuuggguuuu.......................................................................   4       0    seq
    ....................caaaaaguucguuuggguuuuu......................................................................   8       0    seq
    ....................caaaaaguucguuCggguuuuu......................................................................   1       1    seq
    .....................aaaaaguucguuuggguuu........................................................................   3       0    seq
    .....................aaaaaguucguuCggguuuu.......................................................................   1       1    seq
    .....................aaaaaguucguuugUguuuuu......................................................................   1       1    seq
    .....................aaaaaguucguuuggguuuuu......................................................................   52      0    seq
    .....................aaaaaguucguuuggguuuuua.....................................................................   1       0    seq
    ......................aaaaguucguuuggguuuC.......................................................................   2       1    seq
    ......................aaaaguucguuuggguuuuuU.....................................................................   10      1    seq
    ......................aaaaguucguuuggguuuuua.....................................................................   4       0    seq
    ............................................................aaaaUccaaacgaacuuuuug...............................   1       1    seq
    ............................................................aaaacccaaacgaacuuuuugU..............................   1       1    seq
    ...............................................................acccaaacgaacuuuuuggc.............................   1       0    seq
    ......................................................................cgaacuuuuuggcccaacccag....................   1       0    seq
```
